# Identifying transcription factors and microRNAs as key regulators of pathways using Bayesian inference on known pathway structures

**DOI:** 10.1186/1477-5956-10-S1-S15

**Published:** 2012-06-21

**Authors:** Damian Roqueiro, Lei Huang, Yang Dai

**Affiliations:** 1 Department of Bioengineering, University of Illinois at Chicago, Chicago, Illinois, 60607, USA

## Abstract

**Background:**

Transcription factors and microRNAs act in concert to regulate gene expression in eukaryotes. Numerous computational methods based on sequence information are available for the prediction of target genes of transcription factors and microRNAs. Although these methods provide a static snapshot of how genes may be regulated, they are not effective for the identification of condition-specific regulators.

**Results:**

We propose a new method that combines: a) transcription factors and microRNAs that are predicted to target genes in pathways, with b) microarray expression profiles of microRNAs and mRNAs, in conjunction with c) the known structure of molecular pathways. These elements are integrated into a Bayesian network derived from each pathway that, through probability inference, allows for the prediction of the key regulators in the pathway. We demonstrate 1) the steps to discretize the expression data for the computation of conditional probabilities in a Bayesian network, 2) the procedure to construct a Bayesian network using the structure of a known pathway and the transcription factors and microRNAs predicted to target genes in that pathway, and 3) the inference results as potential regulators of three signaling pathways using microarray expression profiles of microRNA and mRNA in estrogen receptor positive and estrogen receptor negative tumors.

**Conclusions:**

We displayed the ability of our framework to integrate multiple sets of microRNA and mRNA expression data, from two phenotypes, with curated molecular pathway structures by creating Bayesian networks. Moreover, by performing inference on the network using known evidence, e.g., status of differentially expressed genes, or by entering hypotheses to be tested, we obtain a list of potential regulators of the pathways. This, in turn, will help increase our understanding about the regulatory mechanisms relevant to the two phenotypes.

## Background

Transcription factors (TFs) and microRNAs are well-known regulators of gene expression. The former bind directly to the regulatory regions of genes whereas the latter regulate the expression of genes at a post-transcriptional stage. Although they have different mechanisms of regulation, evidence suggests that TFs and microRNAs regulate target genes in a coordinated way [1]. In order to facilitate the elucidation of these regulatory mechanisms, several databases have been released based on the analysis of sequence information for predicted regulatory interactions. Backes et al. [2] have compiled a dictionary on microRNAs and their putative pathways based on the enrichment of the predicted microRNAs targets for each pathway in KEGG [3] and TRANSPATH [4]. Le Bechec et al. [5] have created a database (MIR@NT@N) that stores predicted interactions between: a) a TF and its target genes (including microRNAs) and b) microRNAs and their predicted target genes. These databases facilitate the retrieval of regulatory interactions based on a query list as input but the expression data of mRNA and microRNA are not effectively explored. The analysis tool mirConnX [6], recently published, allows the input of concurrent microRNA and mRNA profiling data for an integrative analysis. The targets of TFs and microRNAs are selected based on the association strength between the regulator and its target. In all the above mentioned work, the analysis of the interactions is focused solely on direct targets. In this work we propose a novel integrative method to analyze microRNA and mRNA expression data in conjunction with sequence-based predicted regulators and the structures of existing pathways. We combine all this information into Bayesian networks, which allow the prediction of pathway regulators, not only based on direct targets but also by inference of the most probable effect of the regulators on other downstream genes. (The preliminary results have been presented at the BIBM 2011 conference [7]).

Bayesian networks [8] have been extensively used for the reconstruction of gene networks based on microarray expression data. In this context the goal was the inference of interactions and statistical dependencies among genes. These dependencies were, in turn, used to learn the dynamic structure of a regulatory network [9]. This methodology has been the foundation for numerous algorithmic approaches. In all these cases, the Bayesian network (BN) -or its more generic dynamic counterpart (DBN) - was used as a tool to reverse engineer the gene network, i.e., the interactions between genes were inferred from observational data. In this work, we do not focus on the task of learning the structure of the BN from expression data. Our goal is to use a known network structure, describing interactions between genes and proteins, for Bayesian inference. The network structure can be any experimentally confirmed interaction network (for example, pathways obtained from KEGG [3] or from the Pathway Interaction Database [10]). Due to the fact that only some TFs and no microRNAs are included in the above mentioned pathways, we extend the pathways to contain TFs and microRNAs that are predicted to target nodes in the pathway. We further compute conditional probabilities between the nodes in the extended network using expression data, with the ultimate goal of building a BN for each individual pathway. Finally, these BNs receive as evidence a list of differentially expressed genes and provide as output a ranked list of TFs and microRNAs that best explain the expression level of genes in the network. As a result of this, the output TFs and microRNAs are hypothesized to be putative regulators of the pathway.

## Methods

**Figure 1:**
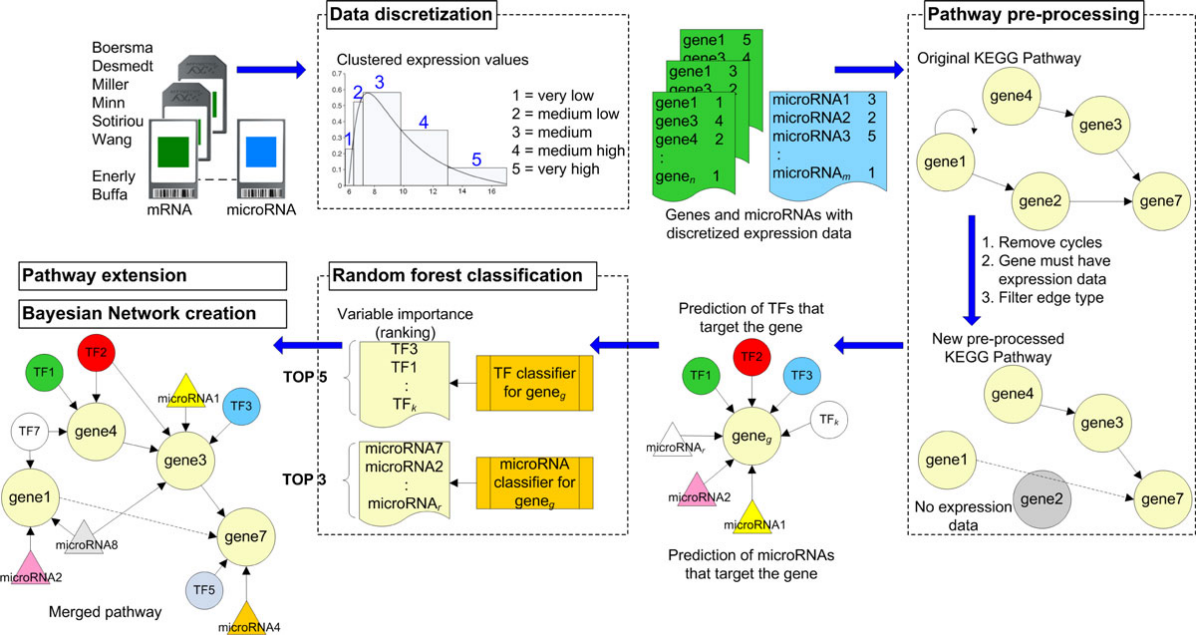
**Flowchart of data integration methodology**. Starting with the processing of raw microarray data, the different analysis steps progress in a clock-wise manner.

We describe our methodology using mRNA and microRNA expression data generated from eight breast tumor studies [11-18]. The patients in these studies were divided into two groups: estrogen receptor positive (ER+) and estrogen receptor negative (ER-). It is important to note that our framework is generic enough so that if multiple datasets of mRNA and microRNA expression data on two specific phenotypes are available, then BNs can be built for inference with the procedure described below. We started by discretizing the expression data of mRNAs and microRNAs from ER+ and ER- tumor microarray profiles. We subsequently obtained the known structure of 34 KEGG pathways and pre-processed them to guarantee that: a) there were no cycles and b) all nodes in the pathway had expression data. For nodes that passed the pre-processing step we proceeded to obtain lists of TFs and microRNAs that are predicted to target the nodes. We then ranked the TFs and microRNAs based on their ability to predict the expression level of a target gene. We obtained one ranking list per gene and expanded the pathways to include the top 5 TFs and top 3 microRNAs for each gene in a pathway. Finally, a BN was created for each extended pathway. Inference was performed by entering, as evidence, the statuses (discrete values) of differentially expressed genes in the pathway. The inference process was performed twice with evidence derived for one phenotype and later with evidence derived from the other phenotype. The marginal probabilities were approximated for all unobserved nodes. From these, the TFs or microRNAs with the largest difference in marginal probabilities between phenotypes were considered the most probable regulators of expression in the pathway. An overview of the entire methodology is illustrated in Figure 1.

### A. Pre-processing of raw microarray data

The raw data from eight studies of ER+/ER- breast tumors [11-18] were downloaded from the Gene Expression Omnibus (GEO). Table 1 provides details of the source of the data and the number of samples for each tumor type. The first six studies contain only mRNA expression profiles whereas the last two (Enerly and Buffa) have concurrent mRNA and microRNA expression profiles on ER+/ER- breast tumors. Herein, we will refer to the datasets using the name provided in Table 1. Supplemental Table S1 in Additional file 1 provides details about the microarray platforms used in these studies.

**Table 1:** Analyzed ER+/ER- expression datasets

Dataset name	Source	Number of samples	
		*ER+*	*ER-*
Boersma	(mRNA) GSE5847 [11]	41	52
Desmedt	(mRNA) GSE7390 [12]	107	51
Miller	(mRNA) GSE3494 [13]	213	34
Minn	(mRNA) GSE2603 [14]	57	42
Sotiriou	(mRNA) GSE2990 [15]	74	24
Wang	(mRNA) GSE2034 [16]	209	77
Enerly	(mRNA) GSE19783 [17]	60	35
Enerly	(microRNA) GSE19536 [17]	60	35
Buffa	(mRNA) GSE22219 [18]	122	79
Buffa	(microRNA) GSE22216 [18]	122	79

Gene expression analysis was performed using packages in Bioconductor [19]. The Robust Multichip Averaging algorithm (RMA) [20] with quantile normalization was used for normalization of the Affymetrix microarrays. Additionally, to minimize the noise level in the subsequent task of data discretization, Affymetrix detection calls were used, only for Affymetrix data, to identify probesets with low or no level of expression.

The raw microRNA data from Enerly were normalized with the RMA algorithm using the AgiMicroRna package [21]. The mRNA data in the Enerly study as well as the mRNA and microRNA data in the Buffa study were already normalized.

**Figure 2:**
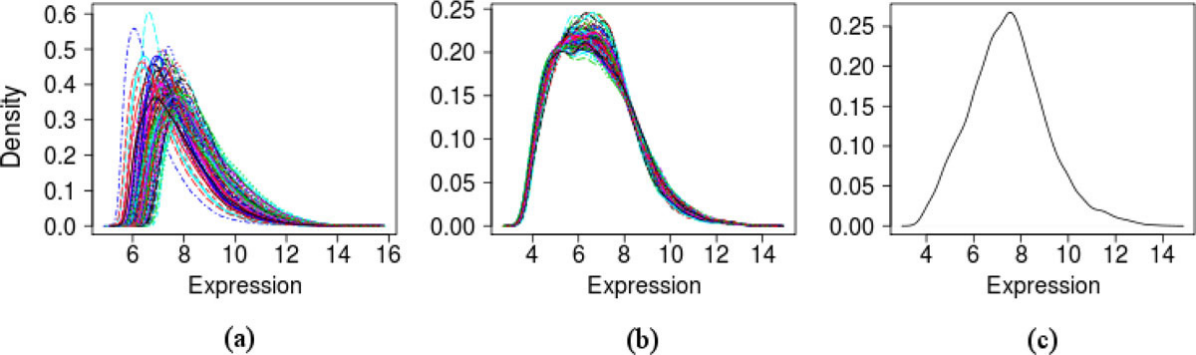
**Density of expression data and discretization**. (a) The raw expression values of all genes in all microarrays of the Boersma dataset, plotted as a density curve. Each curve corresponds to a microarray. (b) The same density curves after the expression values have been normalized. The normalization procedure forces all curves to have the same mean and standard deviation. (c) The density curve of a randomly chosen microarray, after normalization and after removing the probesets marked as "Absent".

Data normalization of each dataset forced its microarrays to have the same empirical distribution of intensities. As an example, the density of expression values of all the microarrays in the Boersma dataset is shown in Figure 2a (before normalization) and Figure 2b (after normalization). In contrast, when only the probesets marked as Present or Marginal were considered, the density function adopted a shape closer to that of a normal distribution (Figure 2c).

The Chip Description Files (CDFs) provided by the manufacturers were used to map probes in the Illumina and Agilent microarrays. A custom CDF was used to map probesets to unique Entrez gene Ids [22] for the Affymetrix microarrays. See Additional file 1, Supplemental Methods, Section A for more details about the pre-processing of microarray data.

### B. Discretization of expression data

A characteristic of BNs is that a node in the network must have distinct (and finite) discrete states. This required a discretization method to convert the expression data obtained from a microarray into discrete values to be fed to the BN. We decided to use 5 states to discretize the expression values of all genes, namely 1 = *very low*, 2 = *medium low*, 3 = *medium*, 4 = *medium high *and 5 = *very high*.

We implemented three discretization methods and compared them in order to determine the most appropriate one for our BNs. The first method was named Sigma-mu and was based on the mean (μ) and standard deviation (σ) of all the expression values of a microarray. The expression level of a gene/microRNA was compared against how many standard deviations away from the mean it was. The five discrete values were assigned as: *very low *and *very high *(≥2σ from μ); *medium low *and *medium high *(≥1σ from μ); and *medium *(< 1σ from μ).

The second method was based on the quantiles of the expression values in a microarray. The density function of the expression values in a microarray was used to obtain estimates of the intervals that accumulated 20%, 40%, 60%, 80% and 100% of the expression values. A discrete value from 1 through 5 was assigned to a gene/microRNA based on the interval on which it fell. Please refer to Additional file 1, Supplemental Methods, Section B for details on these two methods.

The third method we implemented is based on the clustering of expression values of the genes/microRNAs in a microarray. Partition Around Medoids (PAM) was used as our clustering algorithm. The genes/microRNAs whose expression values were clustered in the lowest cluster -cluster 1, corresponding to the lowest expression levels- were discretized as *very low*. Conversely, the genes clustered in the highest cluster (cluster 5) were discretized as *very high*. Genes in clusters 2 and 4 were discretized as *medium low *and *medium high *respectively. Finally, genes in the remaining cluster (3) were discretized as *medium*.

Independent of the discretization method used, probesets marked as "Absent" in Affymetrix microarrays were given a discrete value of *very low*. This process was repeated for all microarrays to yield a discrete value for each gene/microRNA in each microarray.

### C. Differential expression analysis

Determining what genes were differentially expressed in each of the eight datasets had different purposes. Differentially expressed genes in the first six datasets were used to determine the most appropriate discretization method, whereas the differentially expressed genes in the Enerly and Buffa datasets were used as evidence in the Bayesian inference process.

The differential expression analysis was performed on all normalized microarrays. For Affymetrix, a probeset was discarded if it was not marked as "Present" or "Marginal" in more than 85% of the samples in the study, or if the coefficient of variability (CV) of the expression values of the probeset was less than 50% across samples in the study. The limma package [23] with the Benjamini-Hochberg correction for multiple tests was used for differentially expression analysis. The adjusted p-value threshold was set to 0.05.

For the Agilent mRNA chips, the normalized expression data were downloaded from GEO and only the probes with unique Entrez gene Ids were kept. For the Agilent microRNA data, the probes with a detection signal of less than 10% of the samples or not associated with H.sapiens were discarded.

The normalized expression data of Illumina mRNA chips were downloaded from GEO and those probes with unique Entrez gene Ids were retained. Probes with a CV less than 20% were filtered out. For the Illumina microRNA chips, only probes associated with H.sapiens were retained. The differential expression analyses were performed with limma as described above. See Additional file 1, Supplemental Methods, Section C for more details.

### D. Structure pre-processing for KEGG pathways

The KEGG database [3] provides experimental knowledge in many forms, one of them being molecular networks called KEGG pathway maps. For our work, the pathway maps were analyzed as networks, with directed edges between the nodes representing a known interaction. The pathways analyzed were related to signaling (KEGG Ids 04010-04350) and cancer (05200- 05223).

The structure of a pathway including nodes and edges was used as the backbone of a BN. Before the BN could be constructed, a pre-processing step was implemented on the pathway. This pre-processing yielded a new network, based on the original pathway, with the following properties:

• No cycles: The KEGG pathway was transformed into a directed acyclic graph (DAG). Edges that created a loop were discarded.

• Nodes with expression data: The Entrez ID of each node in the pathway was checked against the list of genes that had expression data (10,722 Entrez IDs from our microarray analysis, see Additional file 1, Supplemental Methods, Section B). Nodes with no expression data were removed. The parents and children (if any) of a removed node were updated to include new edges linking them.

• Limited types of interactions: Only the following interactions annotated in a KEGG pathway were taken into consideration: a) gene expression relations: *expression*, *repression *and *indirect effect*; and b) protein-protein interactions: *activation*, *inhibition *and *indirect effect*.

The package KEGGgraph [24] in Bioconductor was used for parsing the raw KEGG Markup Language files.

### E. The predicted targets of transcription factors and microRNAs

Since our goal in implementing a BN for a known pathway is the identification of the set of TFs and microRNAs that are putative regulators of nodes in the pathway, the new network obtained from the previous pre-processing step needed to be expanded to include the TFs and microRNAs that are predicted to target the nodes in the pathway. We followed two different approaches to determine which TFs and microRNAs may target a node in the pre-processed network.

1. TF target prediction. bindSDb [25] is a database we developed to store experimentally proven and predicted transcription factor binding sites. For the prediction portion, the database returns a set of TFs that are predicted to bind to the promoter region of a gene based on sequence analysis. It uses the MATCH [26] algorithm to determine if a TF may bind to the promoter of the gene. Each TF was represented by one or more position weight matrices from TRANSFAC (ver. 2010.1) [27]. In our work, for each gene in a pathway, or protein encoded by a gene, we obtained from bindSDb all the TFs that are predicted to bind to the promoter region of the gene (defined as +2Kb, -2Kb from the transcription start site).

Additionally, we obtained from TRANSFAC the information about the genes that encode the predicted TFs (when available). In this way, each gene in the pathway will be associated with a set of genes whose protein products, i.e. TFs, are predicted to target the gene. If one of the predicted TFs was already present in the pathway, then it was not included as a putative regulator of the gene.

2. microRNA target prediction. All microRNA-gene predictions were downloaded from TargetScan Human release 6.0 [28]. TargetScan is a microRNA target prediction algorithm that searches highly conserved 3'UTR targets for 8-mer and 7-mer sites matching the seed region of microRNAs. We downloaded target predictions for 677 microRNA families, as defined by TargetScan, and obtained a total of 54,479 unique pairs between microRNA family and target gene.

### F. Selection of TFs and microRNAs with Random Forest

The previous step provided a list of predicted TFs and microRNAs targeting each individual gene in a pathway. Ideally, we would expand our pathway by adding incoming edges to a gene from every TF and microRNA in that list. As it will become clear later, this was infeasible especially because of the large number of TFs and microRNAs that may target a gene. Table 2 shows the number of TFs and microRNAs that are predicted to target the genes of three signaling pathways (see Additional file 2 for full details). If a node in a BN has more than 100 parents, we simply cannot maintain its conditional probability table (such a table will consist of 5^100 ^entries). Therefore, it is necessary to limit the number of regulators for each gene. To that respect, we used a machine learning approach to obtain a rank of the TFs and microRNAs that are predicted to target each gene. Based on this ranking a few top regulators of a node will be selected as additional parents of the node. Two classifiers were created with the random forest (RF) classification algorithm [29] on each gene of a pathway based on the expression levels of the associated TFs and microRNAs, respectively. For each classifier, the values of the predictor variables were the discretized expression levels of the TFs (mRNAs' of the encoding genes) or microRNAs. Our ultimate goal was not to find a classifier to predict the expression level of genes but to use RF to measure the importance of each predictor variable. In this manner, for each gene, a group of TFs and microRNAs that could differentiate the expression level of the gene across different microarrays were obtained.

**Table 2:** Number of target TFs and microRNAs

KEGG Id	Pathway name	Number of TFs per node		Number of microRNAs per node	
		*Average*	*Max*	*Average*	*Max*
04010	MAPK signaling pathway	95.3	165	10.7	54
04150	mTOR signaling pathway	90.3	152	17.1	59
04115	p53 signaling pathway	91.8	151	14.2	59

This table shows the number of TFs and microRNAs that target genes in a KEGG pathway. The column *Average *indicates the average number of TFs or microRNAs targeting a gene in the pathway. The maximum number of TFs or microRNAs targeting a gene in that pathway is shown in column *Max*. This is equivalent to the number of incoming edges the node will have if all TFs or microRNAs were added to the pathway.

**Figure 3:**
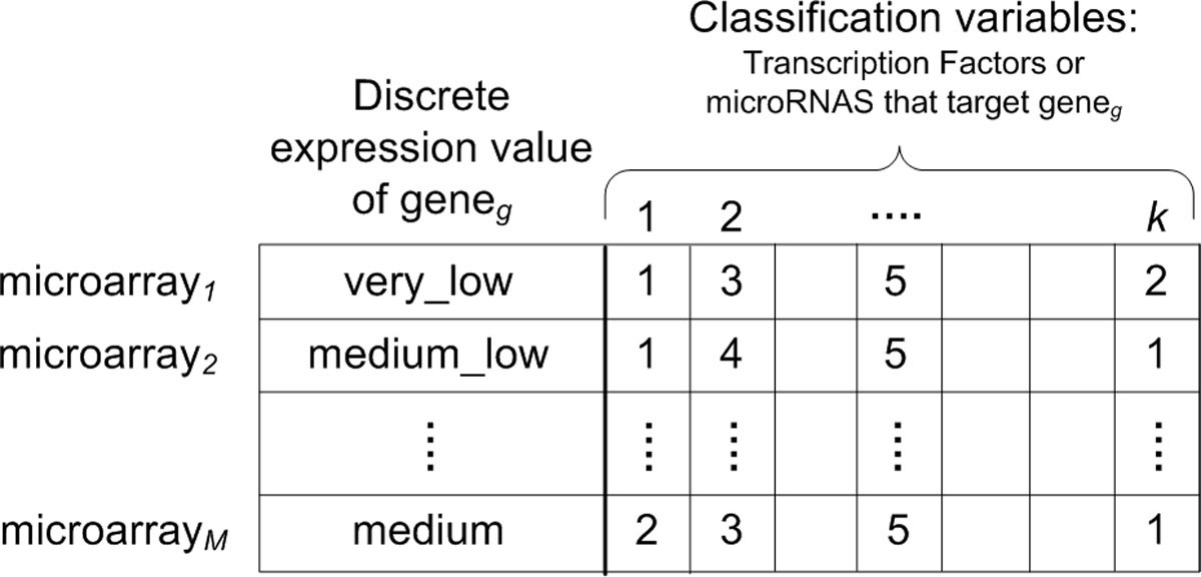
**Data layout for random forest classification**. Layout of the data matrix for gene***_g _***given as input to random forest. The first column indicates the discrete expression value of the gene. The other ***k ***columns correspond to the ***k ***predictors -TFs or microRNAs, one classifier for TFs and another for microRNAs- predicted to target gene***_g_***. The values for each of the columns are also the discrete expression values (as a number between 1 and 5) of the predictors in all microarrays.

The layout of the input data to RF is shown in Figure 3. More specifically, the supervised learning predictor for gene *g *is defined as *T_g _*= (*y_i_*, ***x***_*i*_) with *i *= 1 to *M*, where *M *is the total number of microarrays used in the classifier. For TFs, *M *= 980 (the first six studies listed in Table 1) and for microRNAs, *M *= 296 (Enerly and Buffa datasets). The multi-class response vector ***y ***contains the *M *discrete expression levels of gene *g *in the microarrays. Each vector ***x***_*i *_has values for *k *predictor variables (TFs or microRNAs that target gene *g*), that is, *x_ij _*for *j *= 1 to *k *contains the discrete expression value of predictor *j *in microarray *i*. The values were coded according to the data discretization: from 1 through 5, where 1 = *very low *and 5 = *very high*. For each gene, an ensemble of 2,000 trees (for TFs) and 500 trees (for microRNAs) was created. One third of the variables were randomly chosen at each tree level and one third of the samples were left as out of bag. Variable importance was determined after performing permutations on the trees to assess the change in their predicting power. Each variable was assigned a mean decrease of accuracy score and the ranking of predictor variables for the gene was based on this score. The analysis was implemented with the R package randomForest.

### G. Pathway extension

At this stage, we have all the required information to create a BN for a pathway. The modified pathway obtained after pre-processing in section D was extended to accommodate the TFs and microRNAs ranked in section F. Our RF analysis output two variable importance rankings for each gene: one for the TFs and one for the microRNAs. These rankings list the TFs and microRNAs in decreasing order of the variable importance score assigned to each of them. An extended pathway was then created by connecting each node in the pre-processed pathway with the nodes representing the top 5 TFs and top 3 microRNAs, only if their variable importance score was greater than zero. Note that the same TF may target more than one gene in the pathway. Therefore, the node for the TF was added just once with multiple edges going from this node to different target genes. The same consideration applied to the microRNAs. This newly merged pathway was then fed to the BN process.

### H. Bayesian network construction

Simply put, a BN can be characterized as [30,31]:

• A directed acyclic graph *G *= (***V***, ***E***) where ***V ***is a set of variables and ***E ***is a set of directed edges between the variables.

• Each variable in ***V ***has a finite set of mutually exclusive states.

• For each variable *B *with parents *A_1_*, *A_2_*,..., *A_p _*there is a set of parameter probabilities in the form of conditional probability tables (CPTs) that capture *P*(*B *| *A_1_*, *A_2_*,..., *A_p_*).

The first two items have been addressed in previous sections (pre-processing and discretization). The creation of the CPT for a given node in the pathway was implemented in the following way:

1. If the node did not have any parents, the CPT was basically a vector representing the prior of the node. It was computed by obtaining the frequencies of each discrete value across all the appropriate microarrays (TFs and genes used the first six datasets of Table 1, whereas microRNAs used either the Enerly or Buffa dataset).

2. If the node had parents *A_1_*, *A_2_*,..., *A_p_*, the CPT reflected the probability of all possible combinations of states between the node and its parents. The probability of each possible combination was obtained by counting and then dividing by the total number of observations. A high-dimensional matrix *C *of 5-by-5-by...(*p *+ 1)-times was used to compute the CPT. The matrix *C *was initialized with 1s to assume that each possible combination of states was possible. Then, for each microarray, the discrete expression values of the node and its parents were obtained as a vector ***v ***= [*v_A1_*, *v_A2_*, ..., *v_Ap_*, *v_node_*]. The contents of matrix *C *at the cell *C*[*v_A1_*, *v_A2_*, ..., *v_Ap_*, *v_node_*] were then incremented by one. At the end, each position of *C *was divided by the sum of all elements in *C*. The matter of what set of microarrays to use was resolved in the following way:

• If any of the node's parents *A_1_*, *A_2_*,..., *A_p _*was a microRNA, either the Enerly or Buffa dataset was used.

• Otherwise, the first six datasets listed in Table 1 were used.

This distinction was absolutely necessary. In order to compute the CPT of a node that had at least one microRNA as parent, we needed to process microarrays that had both expression values for genes/TFs as well as microRNAs. Evidently, the CPTs of nodes with a microRNA targeting them were created from fewer observations than nodes whose parents were only TFs or other pathway nodes.

An important aspect of a BN is the evidence, i.e., the values assigned to the observed nodes. For evidence, the 907 differentially expressed genes between ER+ and ER- samples that overlapped as the top 2000-ranked genes in the mRNA-Enerly and mRNA-Buffa datasets were used (See Additional file 1, Table S6). Only those differentially expressed genes that were part of a pathway (not as TFs but as KEGG pathway nodes) were used as evidence.

Once the BN for a pathway was created, we conducted two rounds of inference. Because the CPTs in our BN were created using all the data from ER+ and ER- samples, in order to identify a contrast between these two conditions we subjected the same BN to two different sets of evidence corresponding to two scenarios. In scenario #1, the evidence value assigned to a gene was the median of all the discrete values of that gene corresponding to ER+ samples. Conversely, in scenario #2, the evidence was formed by obtaining the medians of the discrete values in ER- samples. Regardless of which of the two scenarios we are analyzing, for a BN with variables *X_1_*, *X_2_*,..., *X_n+s _*where the evidence ***e ***= [*X_n+1_*, *X_n+2_*,..., *X_n+s_*] and the values of variables *X_1_*, *X_2_*,..., *X_n _*are unobserved, we would like to obtain *P*(*X_1_*, *X_2_*,..., *X_n _*| ***e***). This joint probability is defined as:

(1) $$P\left(X_{1},X_{2},\ldots ,X_{n+s}\right)=\overset{n+s}{\underset{i=1}{\prod }}P\left(X_{i}|parents\left(X_{i}\right)\right)$$

Because the size of the CPT for each variable *X_i _*is exponential on the number of parents of *X_i_*, this computation is prohibitive for large networks. To complicate matters further, we would like an answer to the question: what is the probability of *X_i _*= *x *given the evidence ***e***? This requires the marginalization of *X_i _*from equation (1).

Since exact inference is computationally infeasible, we have to find an approximation to the marginal probability *P*(*X_i _*| ***e***). In our work, this was achieved by using a Gibbs sampler. The marginal probabilities for all unobserved nodes were sampled at a rate of *Q *× number of nodes in the BN, with *Q *= 250. See the Results section for details on how *Q *was computed. The BN creation, Gibbs sampler, inference engine and marginalization of nodes were implemented with the Bayes Net toolbox (BNT) for Matlab [32].

## Results

### Comparison of the three discretization methods

Data discretization has a strong effect over the conditional probabilities assigned to each node in the BNs. Therefore, we conducted a comparison of the three discretization algorithms described in the Methods section to determine the one that was most appropriate to our study.

We compared the discrete values obtained from each method to identify the one that created the largest contrast between the two phenotypes in the data (ER+ vs. ER- in this case). To detect this contrast, we used as reference the genes which we had determined to be differentially expressed in each dataset (see Additional file 1, Table S5). In theory, if a gene is differentially expressed in a dataset, it means that the expression values of the gene in the ER+ samples are different from the expression values of the same gene in ER- samples.

To quantify that difference for a given gene*_k_*, we used the unweighted pair-group method arithmetic averages (UPGMA) between ER+ and ER- samples.

$$\Delta gene_{k}=\frac{1}{\left|ER+||ER-\right|}\underset{x\in ER+}{\sum }\underset{y\in ER-}{\sum }d\left(x,y\right)$$

where:

• *gene_k _*must be differentially expressed in dataset*_d_*

• *ER*+ and *ER*- are the samples for each phenotype

• the distance measure *d*() is simply the absolute value of *x *- *y*, where *x *and *y *are discrete values between 1 and 5.

**Figure 4:**
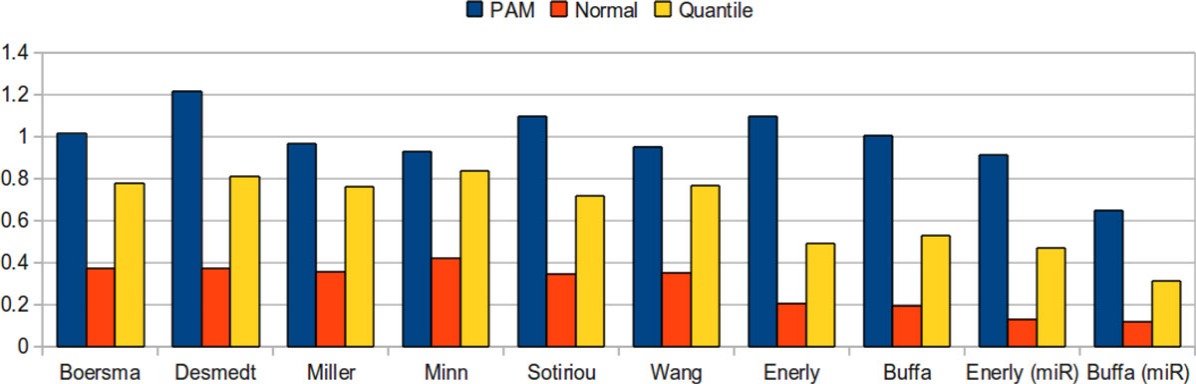
**The average distance of the discretized values of differentially expressed genes between ER+ and ER-, for all datasets**. UPGMA of discrete values in differentially expressed genes between ER+ and ER-. The PAM discretization method provides the largest difference between the two groups.

Figure 4 shows the average difference as defined above for the three methods in all datasets. Clearly, the PAM method obtained the largest difference of discrete values between the two phenotypes. Other evaluation criteria also confirmed that PAM provided the best discrete values among the three methods (see Additional file 1, Supplemental Methods, Section B). Therefore, the following results were derived based on the PAM method for discretization.

### Approximation of marginal probability for Bayesian inference

**Figure 5:**
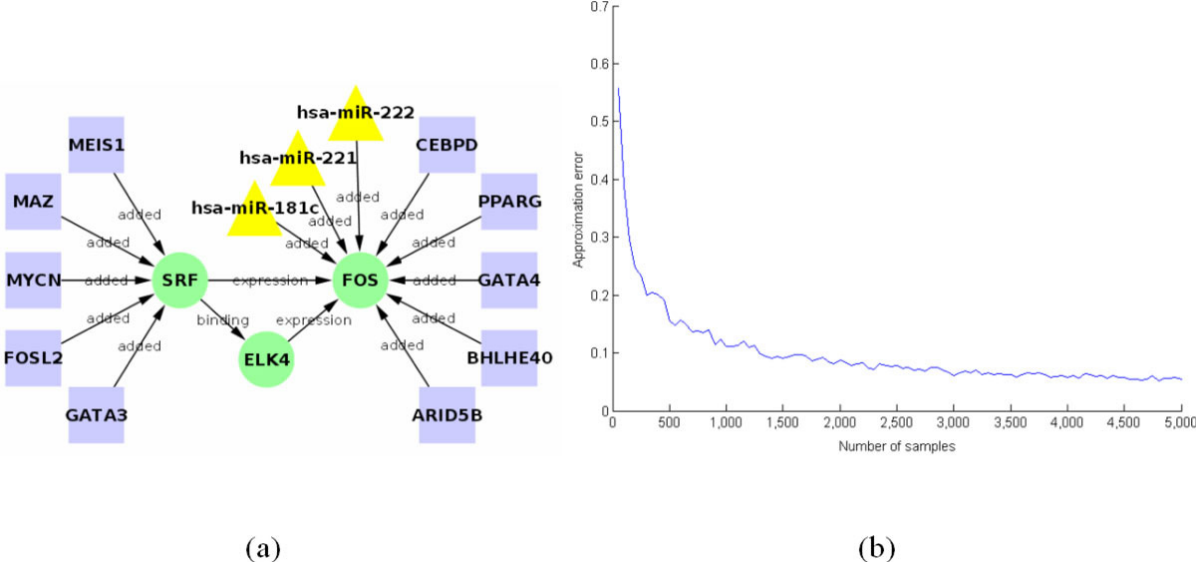
**Toy BN of 16 nodes and its error in approximating marginals using Gibbs sampler**. (a) Network with 16 nodes extracted from the extended MAPK signaling pathway. The three nodes (in green) are the original nodes from the pathway. The TFs (squares) and microRNAs (triangles) that target them are also included. Due to the small size of the network the marginals were computed precisely. (b) Approximation of the marginal after different sample sizes of the Gibbs sampler. It can be seen that after 4,000 samples the error stays below 0.05.

In order to empirically determine the value of *Q*, i.e., the number of samples to draw while using the Gibbs sampler in the estimation of the marginals, we proceeded to create two toy BNs of 16 and 36 nodes. The 16-node network was based on three nodes from the MAPK signaling pathway. These three nodes were subjected to all the steps in our methodology: pathway pre-processing, prediction of target TFs and microRNAs, RF classification and variable importance and, finally, pathway extension. The three pathway nodes (in green) with the TFs (squares) and microRNAs (triangles) that target them are shown in Figure 5a. These two toy BNs were small enough that the full joint probabilities could be computed precisely. Therefore, all marginals were computed in an exact manner. We then approximated the marginals using a Gibbs sampler and the approximation error was determined for different number of iterations of the sampler. For the 16-node BN it can be seen that there is little oscillation of the error, and that after 4,000 samples the error stays below 0.05 (Figure 5b). Our empirical *Q *= 4,000/16 = 250 was used to determine how many samples had to be taken per node. A similar analysis was done with the 36-node network arriving to a similar value of *Q*. For this network we continued testing the number of samples up to 50,000 to show how the approximation error continues to decrease (See Additional file 1, Figures S7 and S8).

### Inference results on the breast cancer data

We have systematically constructed BNs for all the 34 KEGG pathways based on the procedures described in Methods. The number of nodes and edges in the original pathways and the number of nodes and edges in the expanded Bayesian networks are provided in the Additional file 2.

We present our inference results in an attempt at uncovering the relationships among TFs, microRNAs and pathway genes that are associated with ER+ and ER- breast tumors. ER+ and ER- tumors display different molecular patterns in terms of cell differentiation, proliferation, survival, invasion and angiogenesis. Understanding the distinct molecular mechanisms in tumors with different ER status will provide insight into potential novel targets for breast cancer treatment [33].

Each BN of a pathway was given two different sets of evidence corresponding to two scenarios. In scenario #1, the evidence was the discrete values in ER+ samples of the differentially expressed genes. After providing the BN with the evidence we ran the inference process and approximated the marginals for all unobserved nodes. In scenario #2, the same inference process was performed and the marginals were approximated. In this case, the evidence used was the discrete values in ER- samples of differentially expressed genes.

**Figure 6:**
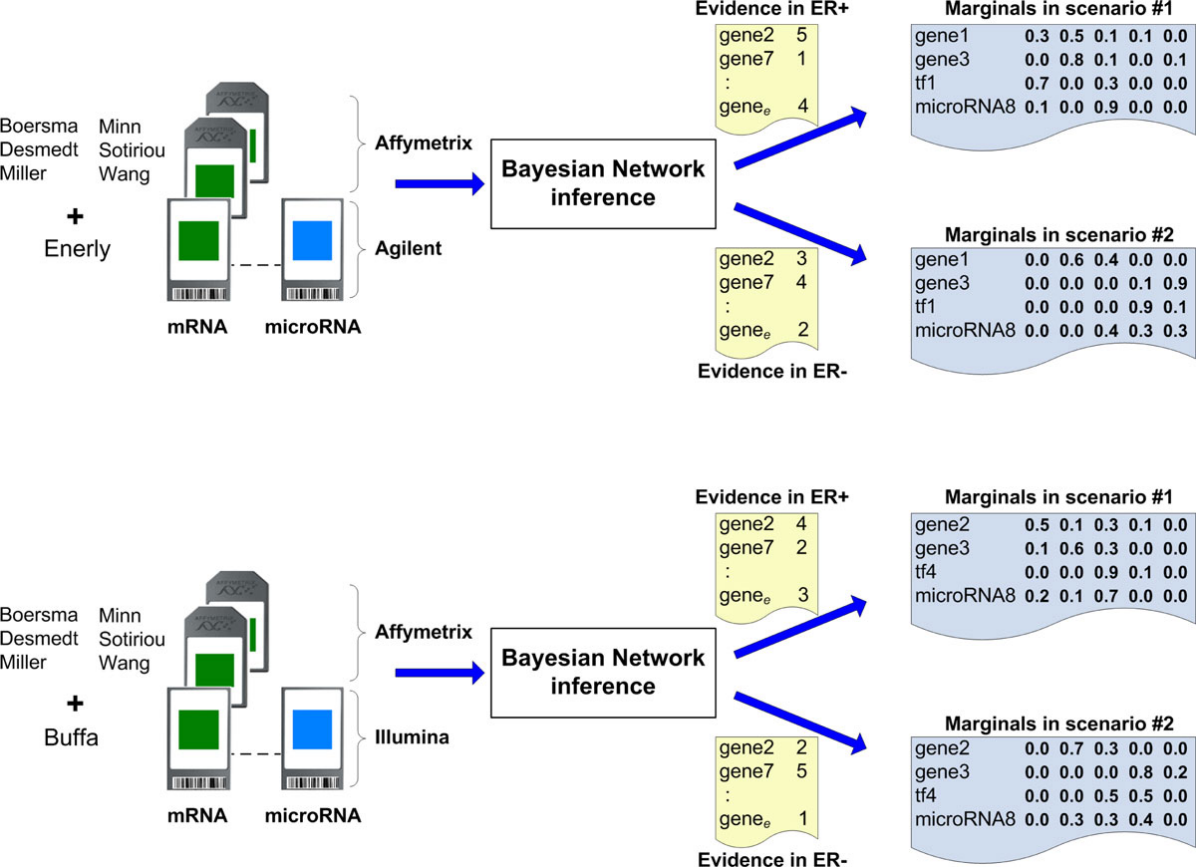
**Inference process to generate results**. For each pathway we generated two BNs: the first one with all mRNA datasets and Enerly and the second one with all mRNA datasets and Buffa. Additionally, each BN received evidence based on two scenarios: the ER+ and ER- inference was run for each scenario to determine changes in the marginal probabilities.

In addition to these two scenarios, we created two BNs for each pathway: one BN using the first 6 datasets + Enerly and another using the first 6 datasets + Buffa. Although many nodes in each BN had the same CPTs, those nodes that had a microRNA as parent had their CPTs derived from a different dataset (either Enerly or Buffa). Our goal in creating these two BNs was to provide further validation to our predictions. If we find a TF or microRNA that our inference process reports as a highly probable regulator, and this coincides in both the Enerly- and Buffa-derived BNs, that provides a greater confirmation that our prediction is plausible. Figure 6 depicts the flowchart of the analysis to create two BNs and to run inference using two scenarios.

When analyzing the results, we decided to focus on nodes that fulfilled any of the following two conditions:

• the node's marginals had one state with a probability larger than 0.8 in scenario #1 and lower than 0.8 in scenario #2 (or vice versa).

• at least one of the node's marginals for one state had a 2-fold variation in probability between scenario #1 and scenario #2, with the resulting probability being larger than 0.5.

There is no particular reason why we chose these threshold values. They are in fact very stringent and served the purpose of providing a reduced set of results that were easy to manually validate against the true KEGG pathway structure.

#### Cell cycle pathway

**Figure 7:**
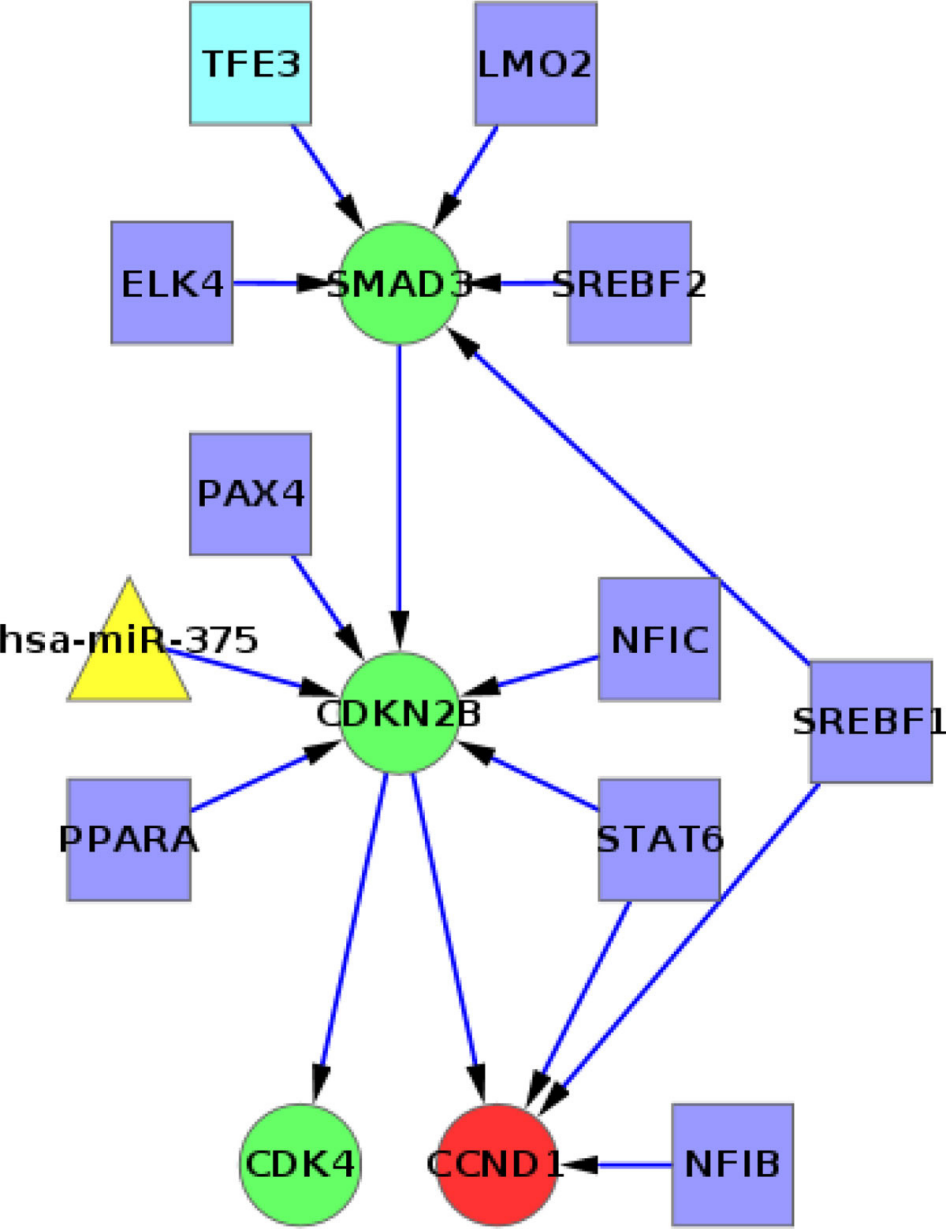
**Analysis of the cell cycle pathway**. Selected nodes from the merged cell cycle pathway. The original nodes in the pathway are in green. The TFs (squares) and microRNAs (triangles) that target them are also included. The differentially expressed gene CCND1 is marked in red and the TF TFE3 (putative regulator) is in light blue According to the pathway definition in KEGG, SMAD3 promotes the expression of CDKN2B and CDKN2B inhibits CCND1 and CDK4. According to our analysis, CCND1 was over-expressed in ER+ samples.

In the cell cycle pathway (KEGG Id 04110) we had 9 differentially expressed genes that were obtained from our differential expression analysis. One of those genes, CCND1 (Cyclin D1), was over-expressed in ER+ samples (Figure 7, marked in red). Being over-expressed in ER+ means that the expression level of CCND1 in ER+ samples was larger than that in ER- samples, in a statistically significant way. Table 3 shows how the discrete value of CCND1 in scenario #1, when the discrete value corresponding to ER+ is used as evidence, is larger than the discrete value in scenario #2, when the discrete value corresponding to ER- is used instead. It goes from *very low *in #2 to *medium *in #1. Table 3 also shows the marginals for a selected group of nodes when the 6 datasets + Buffa were used to create the BN (Table S8 in Additional file 1 shows the marginals for the 6 datasets + Enerly). These marginals, for each scenario, indicate the most probable state in which the expression of a gene, TF or microRNA might be, based on the evidence entered in that scenario.

**Table 3:** Selected probability marginals for the cell cycle pathway (6 datasets + Buffa)

Node	Marginals									
	
		Scenario #1					Scenario #2			
	
	*very low*	*medium low*	*medium*	*medium high*	*very high*	*very low*	*medium low*	*medium*	*medium high*	*very high*
SMAD3	0.29	0.39	0.18	0.09	0.06	0.21	0.31	0.19	0.12	0.16
CDKN2B	0.99		0.01		0.01	0.48	0.11	0.12	0.14	0.14
CCND1			(de)*			(de)				
CDK4	0.01		0.01	0.82	0.16	0.12	0.2	0.18	0.28	0.22
TFE3			0.69	0.31			0.04	0.31	0.65	0.01
LMO2		0.06	0.82	0.12	0.01	0.01	0.14	0.77	0.08	0
ELK4	0.98	0.01		0.01		0.98		0.01		0.01
SREBF2	0.01		0.99			0.09	0.01	0.89	0.01	
PAX4	1.0					1.0				
NFIC	0.22	0.36	0.35	0.07		0.14	0.36	0.29	0.19	0.01
STAT6	0.01	0.01	0.09	0.9				0.09	0.9	0.01
SREBF1				0.98	0.01			0.07	0.9	0.03
NFIB	0.12	0.26	0.32	0.23	0.08	0.13	0.29	0.29	0.21	0.07
PPARA	0.99	0.01				0.98	0.01		0.01	0.01
hsa-mir-375	0.04	0.1	0.11	0.33	0.42	0.04	0.07	0.15	0.33	0.42

*de: the gene is differentially expressed and was used as evidence.

When inspecting the TFs that from sequence analysis and RF we have predicted to target CCND1 directly (NFIB, STAT6, SREBF1) we realize that their marginals are very similar in both scenarios. Because we know that the expression of CCND1 changed between scenarios #2 and #1, we are looking for a TF or microRNA that may also have changed between those scenarios and that may help explain the change in expression for CCND1. Neither of the TFs or microRNAs (not shown in Figure 7) that target CCND1 have a significant change in their marginals between scenarios. The TF TFE3 (transcription factor binding to IGHM enhancer 3) may provide a better explanation of why CCND1 is differentially expressed, even if TFE3 does not target CCND1 directly. In Figure 7 TFE3 is in light blue and targets SMAD3. Between scenarios #2 and #1 we can see (Table 3) that there is more certainty in scenario #1 that SMAD3 is at a lower state (a combined *very low *and *medium low *of 0.29+0.39 = 0.68). This implies a lower level of expression in that scenario (vs. 0.21+0.31 = 0.52 in scenario #2). The marginals have a moderate change from higher expression states in scenario #2 to lower states in #1. This transition is much sharper for Enerly (See Additional file 1, Table S8). In the Cell cycle pathway, SMAD3 promotes the expression of CDKN2B, which in turn regulates the expression of CCND1 and CDK4 by inhibiting them. Our BN simply keeps directed edges between nodes (as in Figure 7) but is not aware of the semantics of each edge (inhibition, expression, and so forth). Nevertheless our results adjust very well to the semantics of the pathway. When SMAD3 switches to a lower state (from scenario #2 to #1), CDNK2B has also a sharp increase of certainty of being in a *very low *expression state (from 0.48 in scenario #2 to 0.99 in scenario #1). Therefore, with a high chance of having low expression of CDKN2B, we also have a high chance of not inhibiting neither CCND1 nor CDK4 and this results in an increase in their expression level (for CCND1, from *very low *in scenario #2 to *medium *in #1; and for CDK4 it goes from a somewhat uncertain state of expression in scenario #2 to a 0.82 certainty of having *medium high *expression level in scenario #1).

Upon reviewing the TFs that are predicted to target SMAD3, we see that TFE3 is the only one with a marked contrast between scenarios. In scenario #2 there is 0.65 probability that its expression is *medium high *but this probability decreases to a 0.31 (more than 2-fold decrease) in scenario #1. This sharp decrease occurs because in scenario #1 there is more certainty of TFE3 being in a *medium *state of expression (0.69 vs. 0.04 in scenario #2). We therefore hypothesize that the transcription factor TFE3 is a key regulator in the Cell cycle pathway when comparing ER- and ER+ samples. We are not implying by any means that TFE3 affects directly the expression of SMAD3 but there is a clear relationship between their changes in expression levels and this allows us to postulate TFE3 as a regulator in the pathway.

In fact, TFE3 is a well-known transcription factor [34] and there is ample evidence of its synergizing effects with SMAD3 to enhance Transformer Growth Factor β (TGF-β) dependent transcription [35,36].

#### p53 signaling pathway

The analysis of the p53 signaling pathway (KEGG Id 04115) provides an example of how to identify a regulator based on direct interactions between the regulator and genes in the pathway. For this pathway, our differential expression analysis reported 8 differentially expressed genes. Very few TFs passed our selection criteria and only one of them overlapped between the Enerly and Buffa datasets. This is the case of STAT5B known as signal transducer and activator of transcription 5B. STAT5B was predicted to target only 2 genes in this pathway: IGFBP3 (insulin-like growth factor binding protein 3) and PERP (p53 apoptosis effector related to PMP-22). These two genes are located in different parts of the pathway and are not directly related to each other. The marginals corresponding to IGFBP3 do not seem to have much of a variation between our two scenarios (Additional file 1, Tables S9 and S10). In contrast, PERP is differentially expressed (under-expressed) in ER+ samples. We can see that the TF STAT5B shifts its certainty of being in a state of *medium low *expression in scenario #2 to a more uncertain state in scenario #1. In fact, in scenario #1 we see an increment in the marginals corresponding to the lowest level of expressions (*very low *and *medium low*) which can be interpreted as a possible decrease of expression of STAT5B from scenario #2 to scenario #1.

STAT5 is one of the seven members of the STAT (signal transducers and activators of transcription) family of TFs and mediates the responses of cytokines, growth factors and hormones [37]. It has been shown that STAT5 regulates apoptosis in a wide range of tumor cells [38]. STAT5A and STAT5B are different proteins encoded by different genes.

PERP, a p53 transcriptional target, is induced specifically during apoptosis but not cell cycle arrest. Downregulation of PERP is associated with the aggressive, monosomy 3-type of uveal melanoma (UM) [39]. It has not been proven that PERP is a direct target of STAT5B [37]. But through our Bayesian inference process we were able to determine that STAT5B (by interacting with PERP) might be a key regulator in the p53 signaling pathway. This result was validated by two BNs constructed with different datasets (Enerly and Buffa)

#### ErbB signaling pathway

The previous results reported only TFs as potential regulators of their pathways. In the BNs of the ErbB signaling pathway (KEGG Id 04012), we identified a microRNA as a putative regulator. In this pathway there were 6 differentially expressed genes. One of them, PLCG2 (phospholipase C, gamma 2 phosphatidylinositol-specific) is under-expressed in ER+ (Additional file 1, Tables S11 and S12). Upon examining the TFs and microRNAs targeting that gene, only the microRNA hsa-miR-135b passed our selection criteria. In scenario #1 of Enerly, hsa-miR-135b reaches a certainty of being at a *medium *expression level (0.81) whereas in scenario #2 there is uncertainty about its level of expression, with higher probabilities in the *very low *and *medium low *states (0.23 and 0.36 respectively). This transition between scenarios #2 and #1 may be seen as an increase in the expression level of the microRNA. Because microRNAs have been shown to negatively regulate the expression of their target genes, if we couple the possible increase in expression of hsa-miR-135b between scenarios #2 and #1 with the fact that the expression of PLCG2 decreases between scenarios #2 and #1, we can propose with higher confidence that hsa-miR-135b is a potential regulator of the ErbB pathway by possibly affecting the expression of PLCG2. In this example, we also had validation of this fact between the Enerly and Buffa datasets.

## Discussion

We proposed an integrative bioinformatics methodology that combines a) the TFs and microRNAs that are predicted to target pathway genes, with b) microarray expression profiles of mRNA and microRNA, in conjunction with c) the known structure of molecular pathways. All these elements were integrated into a probabilistic framework (BN) that was used to make inferences about key TFs and microRNAs as regulators of the pathway. Using the procedures described in our work, one can systematically construct a BN for each individual pathway of interest. We have utilized 8 microarray expression datasets of mRNA and microRNA on ER+ and ER- breast tumors to demonstrate how to use the differentially expressed genes as evidence in order to infer key regulators in the constructed BNs. Another important use of our framework is to propose hypotheses about the expression levels of TFs or microRNAs and their effect on genes. We foresee the researcher posing questions of the form: "What would the expression level of genes *g_1 _*and *g_2 _*be if *microRNA_3 _*is expressed at a *very high *level?"

Several technical issues deserve further investigation. When making inference about the expression level of a gene, TF or microRNA, we would ideally want to obtain the most probable explanation (MPE) given the evidence at hand. This evidence can be tangible, i.e., obtained from a microarray experiment, or, as it was mentioned before, it can be a set of hypotheses that interest us. In either case, an exact solution to the MPE problem in Bayesian inference has proven to be elusive due to the fact that approximating the MPE or finding the *k*-th MPE are both NP-hard problems [31]. Thus, in this work we have decided to use the marginals as a proxy for MPE. In turn, we approximated the marginals for the unobserved nodes using a stochastic sampling algorithm (Gibbs sampler). We plan to improve our methodology by thoroughly examining different importance sampling algorithms that will minimize the variance between the drawn samples and the target distribution [40].

Finally, a self-imposed limitation of our model was the removal of edges that would create cycles in the network. Our next step will be to improve our probabilistic framework to use a dynamic Bayesian network (DBN) that allows for cycles and that better reflects the positive feedback present in many molecular pathways.

## Conclusions

This paper presents a novel approach to the integrative analysis of microRNA and mRNA expression profiles with transcription factors and microRNAs within the context of molecular pathways. We developed a probabilistic framework (Bayesian network) which enables the inference of potential pathway regulators (transcription factors and microRNAs) that are likely causal regulators of the differentially expressed genes in a pathway. Our method may be useful to identify target genes of transcription factors and microRNAs.

## Competing interests

The authors declare that they have no competing interests.

## Authors' contributions

DR and YD conceived the idea and DR implemented the method. DR, LH and YD conducted the data analysis and wrote the manuscript.

## Supplementary Material

Additional file 1 **supplemental-material-B474.doc**. This file contains the Supplemental Methods and Supplemental Results sections. These supplements provide extra details about the analysis steps we followed and the results we obtained. Both these sections contain supplemental tables and figures referenced in the main text. Click here for file

Additional file 2 **supplemental-table-S15.xls**. Contains details about the number of nodes, edges, transcription factors and microRNAs at different stages of the processing of KEGG pathways. Click here for file
