# Supplementary material for: Identifying transcription factors and microRNAs as key regulators of pathways using Bayesian inference on known pathway structures
Source: Proteome Sci. 2012 Jun 21;10(Suppl 1):S15. doi: 10.1186/1477-5956-10-S1-S15 (PMC3380732; doi:10.1186/1477-5956-10-S1-S15)
Supplement: Additional file 1 — supplemental-material-B474.doc. This file contains the Supplemental Methods and Supplemental Results sections. These supplements provide extra details about the analysis steps we followed and the results we obtained. Both these sections contain supplemental tables and figures referenced in the main text. [file 1477-5956-10-S1-S15-S1.pdf]

Supplemental Material

SUPPLEMENTAL METHODS **2**

A. Pre-processing of raw microarray data 2
Table S1. Microarray expression datasets *2*
Table S2. Normalization of microarrays *3*
Figure S1. Expression data and normalization of mRNA datasets *4*
Figure S2. Normalized expression data in combined mRNA-microRNA datasets *5*
B. Discretization of expression data 5
Figure S3. Data discretization overview *6*
B.1 Sigma-mu (σ and μ) *6*
Figure S4. Discretization using sigma-mu (σ and μ) *6*
B.2 Quantiles *6*
B.3 Partition Around Medoids (PAM) *7*
B4. Evaluation Criteria *7*
Table S3. Distance metrics for differentially expressed genes *9*
Table S4. Distance metrics for non-differentially expressed genes *9*
Figure S5. Same vs. different discrete values in differentially expressed genes *10*
Figure S6. Same vs. different discrete values in non-differentially expressed genes (100 runs) *11*
C. Differential expression analysis 11
C1. Protocol *11*
Table S5. Number of probesets/probes after each processing step *13*
C2. Overlaps among differentially expressed gene lists *13*
Table S6. Overlap of differentially expressed genes *14*
Table S7. Genes with opposite differential expression status in Agilent and Illumina microarrays ... *14*

SUPPLEMENTAL RESULTS **15**

Gibbs sampling, approximation of marginal probability for Bayesian inference 15
Figure S7. A toy BN with 36 nodes *15*
Figure S8. The Gibbs sampling error for the 36-node network *15*
Inference results on the breast cancer data 16
Table S8. Selected marginals for the Cell cycle pathway (6 datasets + Enerly) *16*
Table S9. Selected marginals for the p53 signalling pathway (6 datasets + Enerly) *16*
Table S10. Selected marginals for the p53 signalling pathway (6 datasets + Buffa) *16*
Table S11. Selected marginals for the ErbB signaling pathway (6 datasets + Enerly) *17*
Table S12. Selected marginals for the ErbB signalling pathway (6 datasets + Buffa) *17*
Table S13. Selected marginals for the MAPK signalling pathway (6 datasets + Enerly) *18*
Table S14. Selected marginals for the MAPK signalling pathway (6 datasets + Buffa) *18*

Supplemental Methods

A. Pre-processing of raw microarray data

The raw data were downloaded from the Gene Expression Omnibus[1]. Table S1 summarizes information on the microarray platforms used in this work.

Table S1. Microarray expression datasets

Dataset	GEO Accession / Reference	Platform	Probe mapping
Boersma	GSE5847 [11]	Affymetrix Human Genome U133A Array (HG-U133A)	Custom CDF
Desmedt	GSE7390 [12]		
Miller	GSE3494 [13]		
Minn	GSE2603 [14]		
Sotiriou	GSE2990 [15]		
Wang	GSE2034 [16]		
Enerly	GSE19783 [17]	Agilent Whole Human Genome Microarray, 4×44K	Original annotation file
Enerly (miR)	GSE19536 [17]	Agilent Human miRNA Microarray (V2) Kit, 8x15K	
Buffa	GSE22219 [18]	Illumina HumanRef-8 v1.0 expression beadchip	
Buffa (miR)	GSE22216 [18]	Illumina Human v1 MicroRNA expression beadchip	

For the first six datasets a custom CDF was used to find a unique mapping between probesets and Entrez gene Ids. The Affymetrix microarrays used in these studies relied, at the time of their creation, on probes defined upon an earlier genome and transcriptome annotation. This annotation differs significantly from our current knowledge, therefore for our analysis we used a custom CDF that conforms to the latest genome and transcriptome available information. We used a Bioconductor package [19] containing the custom CDF "HGU133A_Hs_ENTREZG" version 13.0 released on July 2010. The Agilent and Illumina microarrays were analyzed using their proprietary annotation files.

The expression data for each study (dataset) were normalized within microarrays of the same study. Normalization varied according to the platform and Table S2 summarizes the steps that were performed:

[1] http://www.ncbi.nlm.nih.gov/geo/

Table S2. Normalization of microarrays

Dataset name	Normalization steps
Boersma<br>Desmedt<br>Miller<br>Minn<br>Sotiriou<br>Wang	Robust Multichip Averaging algorithm (RMA) with quantile normalization.<br>Affymetrix detection calls to identify probesets marked as Present or Marginal (P/M calls)
Enerly	Used normalized data from GEO
Enerly (miR)	RMA algorithm using the AgiMicroRna package [21]
Buffa	Used normalized data from GEO
Buffa (miR)	Used normalized data from GEO

There was a very large variability in the Affymetrix microarrays. This can be seen in Figure S1 (first column) where the densities of expression values for the microarrays are plotted as curves. The second column shows the same curves after normalization. The third column shows, for one randomly chosen microarray in the study, how the normalized density curve changes shape when only the expression values of probesets marked as Present (P) or Marginal (M) are used. This is due to the fact that probesets marked as Absent (A) still have an expression value different from zero. By using P/M calls we followed best practices recommended for Affymetrix microarrays and obtained more reliable data to input to our system.

For the combined mRNA-microRNA studies, only the microRNA expression data in Enerly had to be normalized. The rest of the expression data were originally normalized by the authors of the study. Figure S2 shows the density curves of the expression values in Enerly and Buffa. The first column of the figure has the mRNA data while the second column has the microRNA data.

Figure S1. Expression data and normalization of mRNA datasets

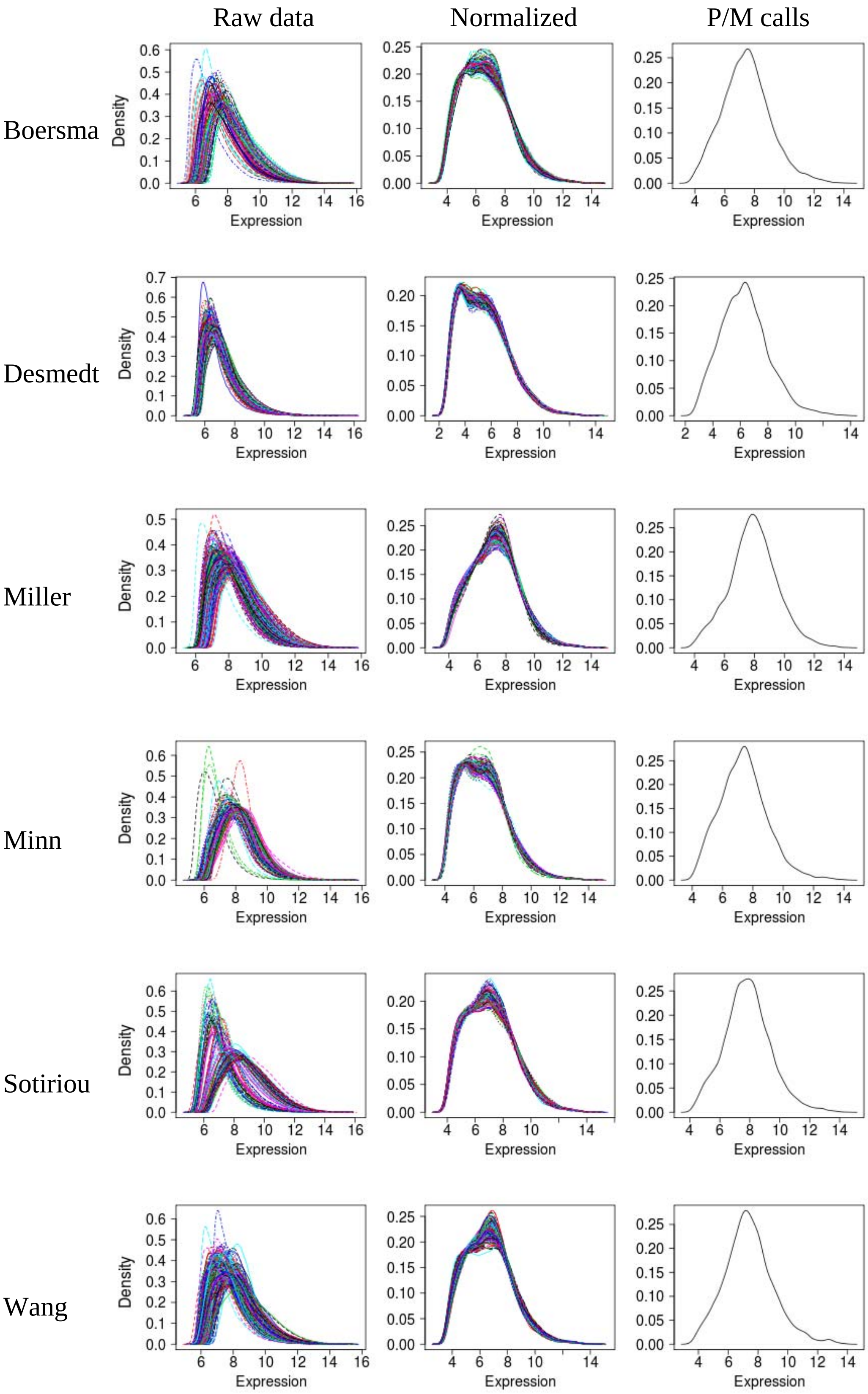

Figure S2. Normalized expression data in combined mRNA-microRNA datasets

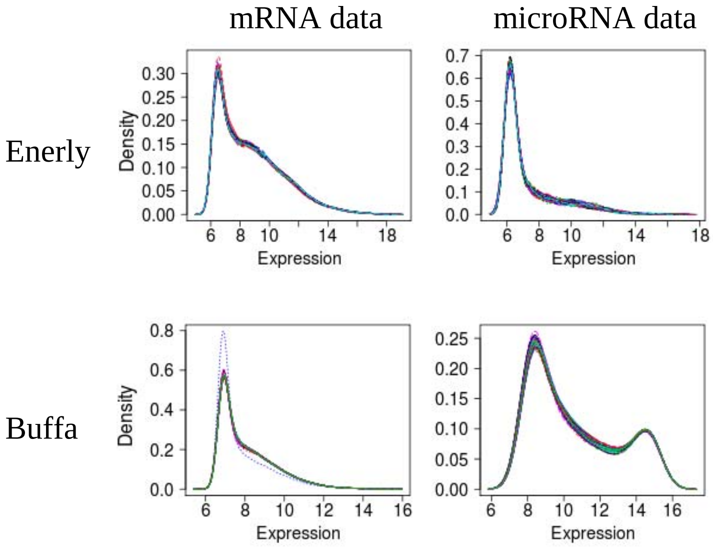

B. Discretization of expression data

From the next section (section C, Differential expression analysis) it will become clear that we could not base our integrative methodology solely on differentially expressed genes. We needed to use all the genes and microRNAs for which we were able to obtain expression data from a microarray. In fact, in order to integrate all eight datasets we had to follow two steps:

1. Determine what genes/microRNAs were common to all eight datasets.
2. Because BNs require discrete states for their nodes, we had to identify a method to convert the expression data obtained from a microarray into discrete values

1. The common genes between the first six datasets and the mRNA-Enerly and mRNA-Buffa datasets were kept. The first six datasets, all of them of the same microarray platform, had 12,025 unique Entrez gene IDs. The mRNA-Enerly and mRNA-Buffa datasets had 17,177 and 15,627 unique genes respectively. A total of **10,722** unique Entrez IDs were common across all mRNA datasets.
The microRNA-Enerly and microRNA-Buffa datasets had 498 and 510 microRNAs respectively with **308** microRNAs that overlapped between both datasets.

2. Regarding data discretization, our choice of how many discrete states to use was five: 1=*very low*, 2=*medium low*, 3=*medium*, 4=*medium high* and 5=*very high*.

In order to determine the best discretization method for our microarray data, we analyzed three discretization algorithms:

1. Sigma-mu (σ and μ)
2. Quantile distribution
3. Partition Around Medoids (PAM)

Figure S3 illustrates the overall approach to data discretization. In the figure we see that the expression data of all the *M* microarrays in a given dataset are discretized one by one.

Figure S3. Data discretization overview

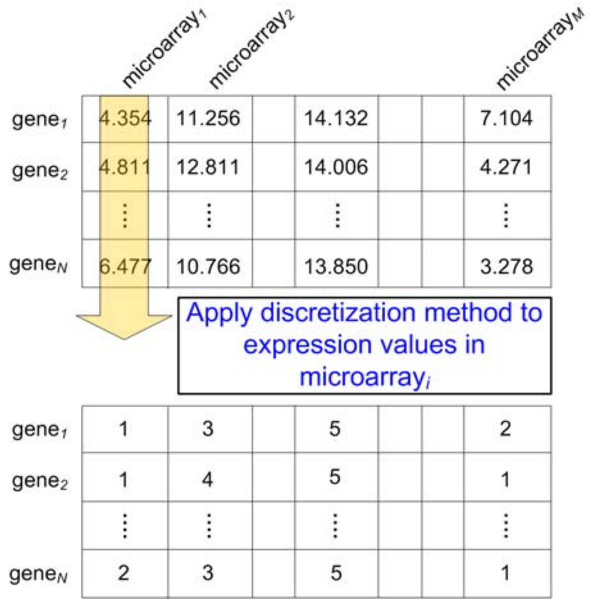

The discretization methods used, as their input, the normalized expression values in the microarrays of a dataset$_d$. The discretization process was repeated for all datasets.

B.1 Sigma-mu (σ and μ)

For each microarray$_i$, the mean and standard deviation of the expression values of genes were obtained. Then, the genes were assigned a discrete value depending on how many standard deviations away they were from the mean (Figure S4). The five discrete values were: *very low*, and *very high* (≥2σ from μ); *medium low* and *medium high* (≥1σ from μ); and *medium* (< 1σ from μ).

Figure S4. Discretization using sigma-mu (σ and μ)

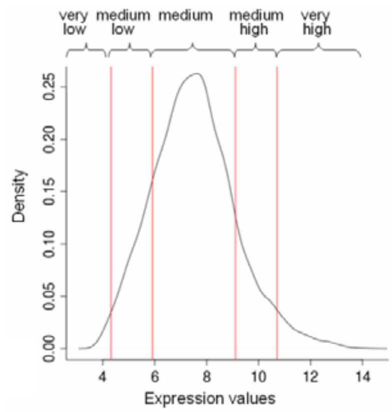

B.2 Quantiles

We applied the quantiles method to obtain estimates of the intervals that accumulated 20%, 40%, 60%, 80% and 100% of the expression values in microarray$_i$. As input we

used the density function of the expression values in the microarray (Figure S4 above). We used the function `quantile` from the R package `stats` that provides sample quantiles. We then discretized the expression values according to the quantile in which they belonged. Quantile 1 (0% to 20%) was assigned a discrete value of *very low*. Quantile 2 (20% to 40%) received a discrete value of *medium low*; all the way to quantile 5 (80% to 100%) which was assigned a discrete value of *very high*.

B.3 Partition Around Medoids (PAM)

The expression values of all the genes in microarray$_i$ were clustered into 5 clusters using PAM (`pam` function from R package `cluster`). The lowest cluster (1) contained genes whose expression values were in the lowest range of expression level. The remaining clusters, 2 through 5 contained genes with higher expression values than those in the previous cluster. Therefore, genes whose expression values were clustered in the lowest and highest end of the spectrum (clusters 1 and 5) were discretized as *very low* and *very high* respectively. Genes in clusters 2 and 4 were discretized as *medium low* and *medium high*. Genes in the remaining cluster (3) were discretized as *medium*.

<u>Note on discretization of Affymetrix microarrays</u>
Regardless of the discretization method used, the above processes were applied only to probesets marked as either “Present” or “Marginal” (P/M). Probesets marked as “Absent” were always given a discrete value of *very low*.

B4. Evaluation Criteria

Each algorithm yielded a set of discrete values for all genes in all microarrays of a dataset. Our goal was to determine which of the sets of discrete values created more contrast between ER+ and ER– samples. Firstly, the contrast was measured for differentially expressed genes and, secondly, for non-differentially expressed genes.

Consider the following example, for dataset$_d$ we have M samples (microarrays). The first s samples correspond to patients categorized as ER+. The remaining M - s samples correspond to ER– patients. Therefore, if gene$_k$ is differentially expressed in dataset$_d$ we can expect the discrete values for gene$_k$ in samples [1...s] to be “significantly different” to the discrete values of the same gene in samples [s + 1…M].

The unweighted pair-group method arithmetic averages (UPGMA) formula below was used to measure the difference in discrete values for a given gene$_k$ in ER+ and ER– samples:

$$\Delta\, gene_k = \frac{1}{|ER+|\,|ER-|} \sum_{x \in ER+} \sum_{y \in ER-} d(x, y)$$

In summary, all the discrete values of gene$_k$ in ER+ samples were compared against the discrete values of gene$_k$ in ER– samples. This process was repeated for all the differentially expressed genes in dataset$_d$, accumulating the $\Delta gene_k$ for all of them.

Our requirements for a good discretization algorithm were the following:

For differentially expressed genes:

1. The sum of all $\Delta gene_k$ should be large: we wanted a discretization algorithm that maximized the distance between discrete values of different phenotypes.
2. The number of genes that get the same discrete value in both phenotypes should be minimized. For example, we wanted to avoid as much as possible a case where $gene_k$ was given a discrete value of, say *very high*, in all *M* microarrays of $dataset_d$. This is an important consideration because even if a gene was determined to be differentially expressed in our analysis, the difference in expression values between phenotypes can be subtle enough that the discretization method could potentially generate the same discrete values for both phenotypes.

Conversely, the same requirements (with opposite criteria) were used for genes that were not differentially expressed.

For the rest of the genes (not differentially expressed):

1. The sum of all $\Delta gene_k$ should be low: in this case, it had to be lower than the value obtained for differentially expressed genes.
2. The number of genes with the same discrete value in both phenotypes should be maximized: it is fair to say that if the gene is not differentially expressed, there should not be much variation between phenotypes.

The sum of all $\Delta gene_k$ per dataset was divided by the number of genes to obtain an average $\Delta gene_k$. If the discrete values of $gene_k$ were the same in all *M* microarrays, we counted it as *same*. Otherwise, we counted it as *different*. These counts as well as the average $\Delta gene_k$ can be seen as columns in Tables S3 and S4.

Computing these values for the differentially expressed genes was straightforward and the results are shown in Figure S5. But obtaining these values for the rest of the genes required taking random samples of genes (not differentially expressed), computing the sum of all $\Delta gene_k$, obtaining the counts *same* and *different* and repeating the process for 100 iterations. For each dataset the sample size *r* differed based on the number of differentially expressed genes in it. For example, in the Boersma dataset $r = 690$ (See Table S5 in the next section).
After 100 iterations, the counts were averaged and the results for non-differentially expressed genes can be found in Table S4.

Table S3. Distance metrics for differentially expressed genes

	PAM			Sigma-mu			Quantile		
**Dataset**	$\Delta gene_k$	*same*	*diff*	$\Delta gene_k$	*same*	*diff*	$\Delta gene_k$	*same*	*diff*
Boersma	1.013	0	690	0.373	41	649	0.780	17	673
Desmedt	1.219	0	1672	0.372	50	1622	0.811	10	1662
Miller	0.967	0	1521	0.356	50	1471	0.763	29	1492
Minn	0.929	0	1481	0.420	60	1421	0.836	14	1467
Sotiriou	1.099	0	1341	0.347	116	1225	0.718	39	1302
Wang	0.954	0	1834	0.353	42	1792	0.766	23	1811
Enerly	1.098	0	3808	0.207	1130	2678	0.492	338	3470
Buffa	1.007	1	4722	0.195	840	3883	0.528	115	4608
Enerly (miR)	0.912	0	60	0.131	36	24	0.469	10	50
Buffa (miR)	0.650	14	136	0.118	58	92	0.313	22	128

The column $\Delta gene_k$ contains the average UPGMA for all the genes analyzed. The column *same* is the count of genes for which the discrete values were the same across all samples of both phenotypes. The column *diff* indicates the count of genes for which at least one discrete value was different between phenotypes. The addition of *same* + *diff* for each dataset is equal to the number of differentially expressed genes in that dataset (see Table S5 in the next section)

When comparing the counts of *same* vs. *different* obtained for differentially expressed genes we can see that PAM outperformed the other two methods. This was of outmost importance to us because the differentially expressed genes in Enerly and Buffa were used as evidence in the BN inference. Figure S5 shows these counts as percentages.

Table S4. Distance metrics for non-differentially expressed genes

	PAM			Sigma-mu			Quantile		
**Dataset**	$\Delta gene_k$	*same*	*diff*	$\Delta gene_k$	*same*	*diff*	$\Delta gene_k$	*same*	*diff*
Boersma	0.570	143.5	546.5	0.326	173.1	516.9	0.386	221.9	468.1
Desmedt	0.700	218.9	1453.1	0.304	309.6	1362.4	0.343	478.8	1193.2
Miller	0.546	260.5	1260.5	0.283	366.1	1154.9	0.342	466.4	1054.6
Minn	0.487	287.7	1193.3	0.341	287.0	1194.0	0.382	441.2	1039.8
Sotiriou	0.690	209.2	1131.8	0.298	332.4	1008.6	0.360	416.5	924.5
Wang	0.517	279.6	1554.4	0.314	349.0	1485.0	0.352	525.5	1308.5
Enerly	0.858	0.3	3807.7	0.175	1156.7	2651.3	0.346	354.6	3453.4
Buffa	0.772	0.0	4723.0	0.167	844.7	3878.3	0.402	125.3	4597.7
Enerly (miR)	0.853	0.1	59.9	0.079	43.5	16.5	0.365	6.4	53.6
Buffa (miR)	0.534	16.5	133.5	0.124	45.1	104.9	0.285	15.5	134.5

The definitions for the columns are the same as in Table S3. The counts *same* and *diff* are averages of the 100 runs. The addition of *same* + *diff* is equal to sample size of the

dataset, which coincides with the number of differentially expressed genes in that dataset (see Table S5)

Figure S5. Same vs. different discrete values in differentially expressed genes

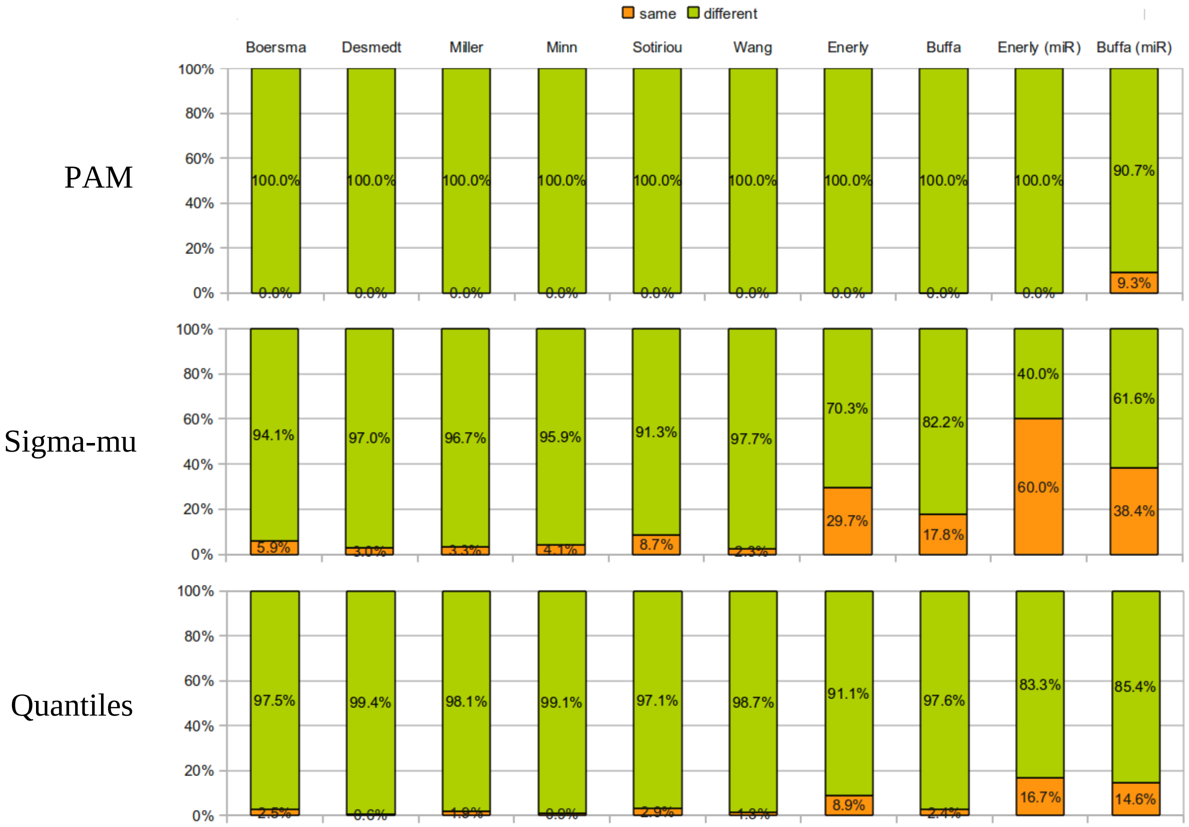

For the non-differentially expressed genes, the Quantiles method performed better than the other two. Because the genes were not differentially expressed, we expected to see a higher value for *same* and a smaller value for *different*. Figure S6 shows the results for the non-differentially expressed genes.

Surprisingly, Sigma-mu performed very poorly in both cases. When looking at the shape of the density functions in Figure S1, our intuition suggested that this method would be appropriate. But under close examination we can see there is a positive skew in those density functions which, although not as prominent as in Figure S2, it makes this discretization method to perform badly in comparison to PAM and Quantiles.

Figure S6. Same vs. different discrete values in non-differentially expressed genes (100 runs)

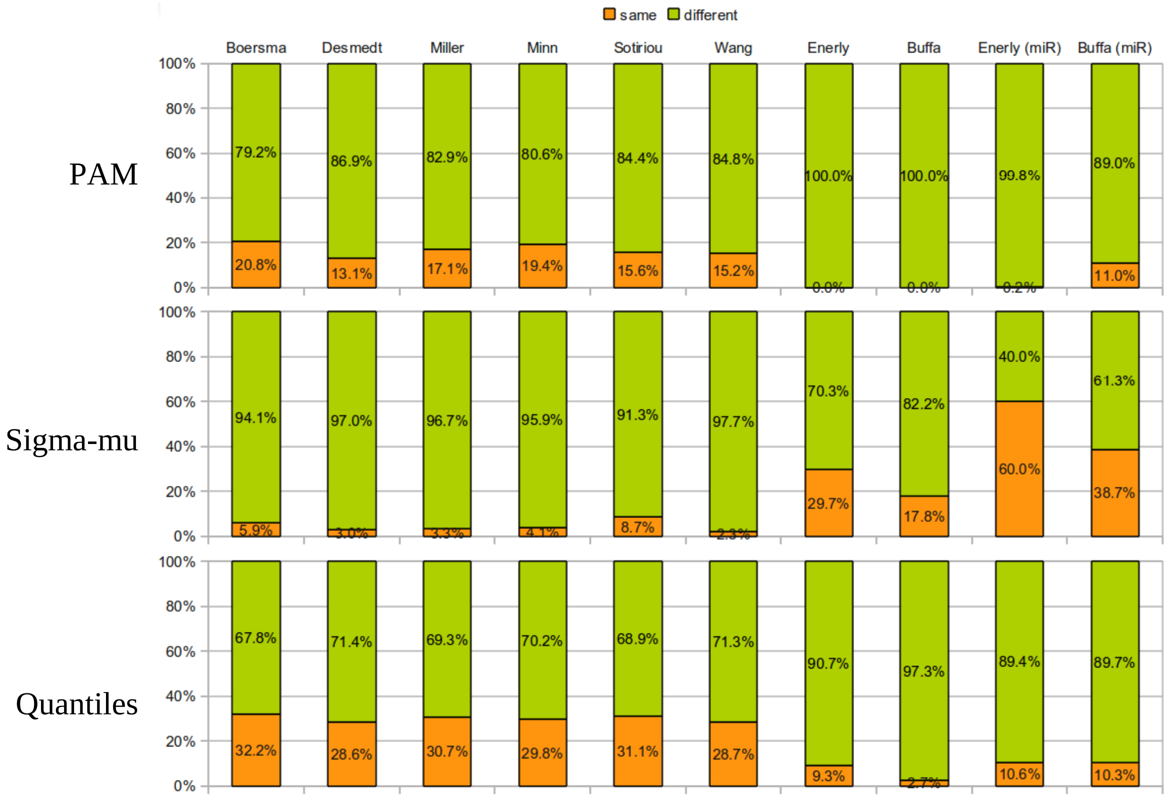

C. Differential expression analysis

C1. Protocol

Lists of differentially expressed genes between ER+ and ER– samples were obtained for all datasets. These differentially expressed genes were used in two different contexts:

- Differentially expressed genes obtained from the first six datasets were used to evaluate different discretization methods (See previous section B in this Supplemental file)
- Differentially expressed genes obtained from the Enerly and Buffa datasets were used as evidence in the BN inference process.

The remainder of this section provides technical details about the process we followed to obtain differentially expressed genes in each microarray platform. We close this analysis by comparing lists of differentially expressed genes obtained in different datasets to show that they do not overlap well.

For Affymetrix (all mRNA data)

1. Use the Wilcoxon signed rank-based gene expression presence/absence detection algorithm from the affy package in R Bioconductor. [function `mas5calls`]
2. Discard probesets not marked as Present or Marginal in 85% of samples.

3. Obtain coefficient of variability (CV) for probesets [CV = standard_deviation/mean]
4. Discard probesets with CV < 0.5 across samples
5. Differential expression using limma package in R Bioconductor [functions `lmFit` and `eBayes`]
6. Adjust p-values using Benjamini–Hochberg correction [function `topTable`]
7. Report probesets with adjusted p-value < 0.05

For Agilent (mRNA)

1. Discard probes that are not associated to an Entrez gene Id(s)
2. Keep the probe with maximum variance across all samples if a gene has multiple probes
3. Follow steps 5 through 7 for the Affymetrix data

For Agilent (microRNA)

1. Discard probes with detection signal in <10% samples or not associated with H.sapiens
2. Follow steps 5 through 7 for the Affymetrix data

For Illumina

1. Discard probes that are not associated to an Entrez gene Id(s)
2. Keep the probe with maximum variance across all samples if a gene has multiple probes
3. Discard probes with CV < 0.20 across samples
4. Follow steps 5 through 7 for the Affymetrix data

The following table summarizes the steps mentioned above and shows the different number of probesets that remained after each processing step. The column **Original** lists the number of probeset/probes after removing those not associated to an Entrez gene Id. The **Filtering method** column indicates how the probes were further filtered.

Table S5. Number of probesets/probes after each processing step

Dataset	Number of probeset/probes				
	**Original**	After P/M calls	Filtering method*	After filtering	Differentially expressed
Boersma	12,133	5,245	1	2,604	690
Desmedt	12,133	6,227	1	3,095	1,672
Miller	12,133	5,932	1	2,952	1,521
Minn	12,133	5,639	1	2,805	1,481
Sotiriou	12,133	6,355	1	3,160	1,341
Wang	12,133	5,753	1	2,859	1,834
Enerly	41,094	–	2	17,117	3,808
Enerly (miR)	729	–	3	498	60
Buffa	24,385	–	2,4	12,501	4,723
Buffa (miR)	735	–	2,5	488	150

*Filtering method (key):

1. Discard probesets with CV < 0.5 across samples
2. For genes with multiple probes, only the probe with maximum variance across all samples was kept.
3. Probes with detection signal in less than 10% of the samples or probes not associated with H.sapiens were discarded.
4. Discard probesets with CV < 0.2 across samples.
5. microRNA probes not associated with H.sapiens were discarded

C2. Overlaps among differentially expressed gene lists

We identified discrepancies when analyzing the lists of differentially expressed genes that were obtained for each dataset. Although the patients in these different studies were divided according to two distinct phenotypes such as ER+ and ER– the differentially expressed genes across studies did not have a large overlap.

For each dataset we ranked the genes according to an ascending order of adjusted p-value. We examined the overlap across multiple datasets for all the differentially expressed genes as well as the overlap for the top 100-ranked genes, 200-ranked genes, and other top rankings. Additionally, we wanted to determine if a gene was reported with a different status in two datasets, e.g. as down-regulated in ER+ samples of one dataset and reported as up-regulated in ER+ of a different dataset. Table S6 shows the details.

Table S6. Overlap of differentially expressed genes

	Overlap of 6 Affymetrix Datasets	Overlap of Enerly and Buffa mRNA datasets
All genes	150	2172
Different status	0	4
Ranking < 100	4	36
Ranking < 200	7	70
Ranking < 500	37	197
Ranking < 1000	116	421
Ranking < 2000	150	907

It can be seen that only 4 genes overlap when taking the top 100-ranked differentially expressed genes in the first 6 Affymetrix datasets. This was a clear sign, early on, that our integrative analysis could not simply rely on differentially expressed genes. As was mentioned before, for our Bayesian inference we used all genes in the microarrays regardless if they were differentially expressed or not.
The information about differentially expressed genes in each of the six Affymetrix datasets was nonetheless useful as was illustrated in the previous section (Section B, Discretization of expression data)

When comparing the Enerly and Buffa datasets, there were 907 genes that overlapped among the top 2000-ranked differentially expressed genes. These 907 genes were used as evidence for the BN inference process.
In these two datasets there are four differentially expressed genes with opposed expression statuses (Table S7). These four genes were discarded from our analysis.

Table S7. Genes with opposite differential expression status in Agilent and Illumina microarrays

Entrez Id	Gene symbol and description	ER+ status in Enerly	ER+ status in Buffa
10270	AKAP8 A kinase (PRKA) anchor protein 8	down	up
29964	PRICKLE4 prickle homolog 4	up	down
128178	EDARADD EDAR-associated death domain	down	up
374655	ZNF710 zinc finger protein 710	down	up

Supplemental Results

Gibbs sampling, approximation of marginal probability for Bayesian inference

Figure S7. A toy BN with 36 nodes

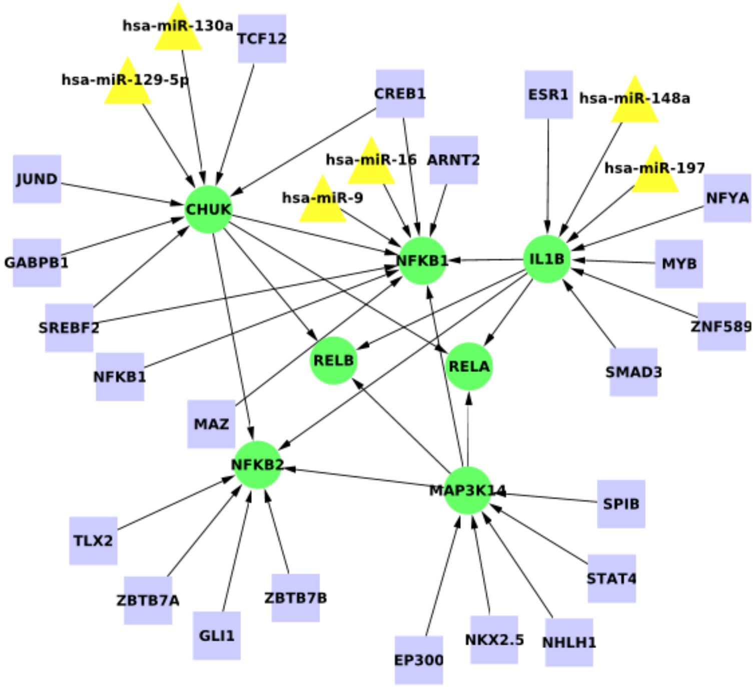

Figure S8. The Gibbs sampling error for the 36-node network

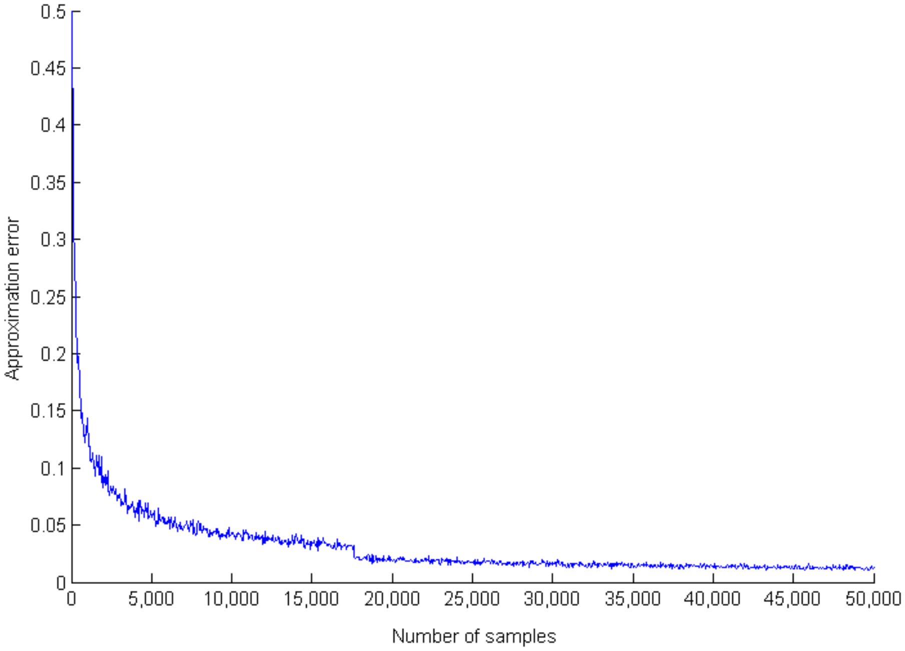

Inference results on the breast cancer data

Table S8. Selected marginals for the Cell cycle pathway (6 datasets + Enerly)

Node	Marginals									
	Scenario #1					Scenario #2				
	very low	*medium low*	*medium*	*medium high*	*very high*	*very low*	*medium low*	*medium*	*medium high*	*very high*
SMAD3	0.14	0.81	0.05	0	0	0.2	0.63	0.08	0.03	0.06
CDKN2B	0.96	0.01	0.01	0.01	0.01	1	0	0	0	0
CCND1					(de)*				(de)	
CDK4	0.01	0.01	0	0.97	0.02	0	0	0	0.68	0.32
TFE3			0.64	0.36				0.86	0.13	0.01
LMO2		0.02	0.92	0.06			0.02	0.93	0.06	
ELK4	0.99	0.01	0.01			0.97	0.01		0.01	0.01
SREBF2			1.0			0.02		0.98		
PAX4	1.0					1.0				
NFIC	0.12	0.34	0.47	0.07		0.2	0.33	0.41	0.06	
STAT6			0.07	0.94				0.08	0.91	0.01
SREBF1			0.01	0.99	0.01			0.01	0.99	0.01
NFIB	0.1	0.27	0.28	0.26	0.09	0.12	0.23	0.33	0.23	0.1
PPARA	0.98	0.01		0.01		1.0				
hsa-mir-375	0.3	0.18	0.16	0.12	0.24	0.37	0.19	0.14	0.13	0.18

*de: indicating that the gene is differentially expressed and was used as evidence.

Table S9. Selected marginals for the p53 signalling pathway (6 datasets + Enerly)

Node	Marginals									
	Scenario #1					Scenario #2				
	very low	*medium low*	*medium*	*medium high*	*very high*	*very low*	*medium low*	*medium*	*medium high*	*very high*
STAT5B	0.16	0.84				0.24	0.73	0.02		
PERP			(de)*						(de)	
IGFBP3	0.23	0.23	0.2	0.14	0.21	0.2	0.2	0.23	0.15	0.21

*de: indicating that the gene is differentially expressed and was used as evidence.

Table S10. Selected marginals for the p53 signalling pathway (6 datasets + Buffa)

Node	Marginals									
	Scenario #1					Scenario #2				
	very low	*medium low*	*medium*	*medium high*	*very high*	*very low*	*medium low*	*medium*	*medium high*	*very high*
STAT5B	0.24	0.74	0.02	0.01		0.12	0.88	0.01		
PERP	(de)*							(de)		
IGFBP3	0.2	0.18	0.21	0.17	0.23	0.15	0.2	0.23	0.23	0.19

*de: indicating that the gene is differentially expressed and was used as evidence.

Table S11. Selected marginals for the ErbB signaling pathway (6 datasets + Enerly)

Node	Marginals									
	Scenario #1					Scenario #2				
	very low	***medium low***	***medium***	***medium high***	***very high***	***very low***	***medium low***	***medium***	***medium high***	***very high***
hsa-miR-135b	0.04	0.14	0.81		0.01	0.23	0.36	0.34	0.02	0.04
PLCG2			(de)*						(de)	

*de: indicating that the gene is differentially expressed and was used as evidence.

Table S12. Selected marginals for the ErbB signalling pathway (6 datasets + Buffa)

Node	Marginals									
	Scenario #1					Scenario #2				
	very low	***medium low***	***medium***	***medium high***	***very high***	***very low***	***medium low***	***medium***	***medium high***	***very high***
hsa-miR-135b		0.01	0.03	0.82	0.14		0.04	0.05	0.71	0.19
PLCG2				(de)*						(de)

*de: indicating that the gene is differentially expressed and was used as evidence.

In addition to the results mentioned in the main text, here we detail other findings obtained for the MAPK signaling pathway which are also related to the TF STAT5B identified in the p53 signaling pathway.

MAPK signaling pathway (KEGG Id 04010)
As it was the case for the p53 signaling pathway, here we also had the chance to identify a regulator based on direct interactions between the regulator and genes in the pathway. We had 15 differentially expressed genes reported for this pathway. Here too, our strict selection criteria yielded few TFs (only 5 in Enerly). Yet, one of them, STAT5B might be considered a regulator for this pathway based on our results.
We predicted 6 target genes of STAT5B: MAP3K12, NFKB2, RRAS2, FGF23, MAPK10 and PTPRR. These genes had no edges connecting them directly in the pathway. The marginals corresponding to the last 3 genes do not vary much between scenario #1 and #2 (Supplemental Tables S13 and S14). In contrast, the first 3 genes do show a difference between the scenarios and MAP3K12 is in fact differentially expressed and up-regulated in ER+ samples. The TF STAT5B shifts its certainty of being in state *medium low* in scenario #2 to a more uncertain state in scenario #1. In scenario #1 we see an increment in the marginals corresponding to higher levels of expression with respect to scenario #2. The fact that STAT5B may slightly increase its expression in scenario #1 provides a better explanation of why NFKB2 and RRAS2 also have a shift in marginals towards higher expression states from scenario #2 to #1. Therefore, we postulate that STAT5B is a potential regulator of the MAPK signaling pathway in breast cancer when comparing ER+ and ER– patients. Because STAT5B showed to be of importance in two different pathways, this reinforces our hypothesis that STAT5B is a key regulator in breast cancer patients.

Table S13. Selected marginals for the MAPK signalling pathway (6 datasets + Enerly)

Node	Marginals									
	Scenario #1					Scenario #2				
	very low	*medium low*	*medium*	*medium high*	*very high*	*very low*	*medium low*	*medium*	*medium high*	*very high*
STAT5B	0.22	0.64	0.09	0.03	0.01	0.1	0.88	0.01	0.01	
MAP3K12			(de)*			(de)				
NFKB2	0.18	0.22	0.22	0.2	0.18	0.74	0.09	0.06	0.06	0.05
RRAS2	0.4	0.14	0.16	0.14	0.16	0.78	0.07	0.06	0.06	0.04
PTPRR	0.27	0.17	0.2	0.21	0.15	0.17	0.18	0.18	0.23	0.24
FGF23	0.15	0.24	0.18	0.2	0.23	0.19	0.2	0.19	0.2	0.21
MAPK10	0.17	0.22	0.23	0.22	0.16	0.18	0.15	0.24	0.2	0.23

*de: indicating that the gene is differentially expressed and was used as the evidence.

Table S14. Selected marginals for the MAPK signalling pathway (6 datasets + Buffa)

Node	Marginals									
	Scenario #1					Scenario #2				
	very low	*medium low*	*medium*	*medium high*	*very high*	*very low*	*medium low*	*medium*	*medium high*	*very high*
STAT5B	0.35	0.53	0.08	0.04	0.01	0.23	0.68	0.05	0.04	
MAP3K12					(de)*		(de)			
NFKB2	0.18	0.21	0.2	0.23	0.18	0.24	0.16	0.18	0.19	0.22
RRAS2	0.17	0.2	0.2	0.23	0.2	0.83	0.03	0.1	0.03	0.02
PTPRR	0.18	0.15	0.24	0.23	0.19	0.17	0.19	0.23	0.2	0.2
FGF23	0.22	0.21	0.19	0.2	0.18	0.2	0.23	0.17	0.21	0.2
MAPK10	0.14	0.2	0.19	0.23	0.23	0.18	0.17	0.2	0.19	0.26

*de: indicating that the gene is differentially expressed and was used as the evidence.
